# Association of MRI indexes of glymphatic system with brain atrophy and cognitive impairment in cerebral small vessel disease

**DOI:** 10.1016/j.nicl.2026.103951

**Published:** 2026-01-18

**Authors:** Lulu Ai, Zhiwei Li, Chaojuan Huang, Xia Zhou, Xiaoqun Zhu, Qiaoqiao Xu, Zhongwu Sun

**Affiliations:** a Department of Neurology, The First Affiliated Hospital of Anhui Medical University, Hefei, China; b Department of Neurology, The Third Affiliated Hospital of Anhui Medical University (Hefei City First People’s Hospital), Hefei, China

**Keywords:** Cerebral small vessel disease, Cognitive impairment, Gray matter volume, Glymphatic system

## Abstract

**Background and objective:**

The glymphatic system constitutes a brain-wide perivascular network responsible for brain metabolic waste removal, which may underlie pathogenesis in cerebral small vessel disease (CSVD). This study aimed to explore the associations of glymphatic function, assessed using multi-modal MRI indices, and both brain atrophy and cognitive impairment in CSVD.

**Methods:**

The study included 160 participants comprising 120 patients with CSVD, including 52 without cognitive impairment (CSVD-NCI) and 68 with mild cognitive impairment (CSVD-MCI), alongside 40 healthy controls (HCs). All participants underwent neuropsychological and multi-modal neuroimaging assessments. Glymphatic function was assessed using four complementary MRI indices: choroid plexus (CP) volume, perivascular space (PVS) volume fraction, free water in white matter (FW-WM) fraction, and diffusion tensor image analysis along the perivascular space (DTI-ALPS) index. Gray matter volume (GMV) was evaluated via voxel-based morphology (VBM) analysis. Partial correlation and mediation analyses explored the relationships among glymphatic function, brain structure and cognitive performance.

**Results:**

Compared to HCs, CSVD-MCI patients showed increased CP volume, FW-WM fraction, BG/putamen-PVS volume, and reduced DTI-ALPS index, accompanied by multifocal gray matter atrophy involving temporal and frontal regions. Advanced age was associated with increased CP and BG-PVS volume, but decreased DTI-ALPS index. A main effect of sex was observed, where males exhibited larger BG-PVS and FW-WM fraction, with lower DTI-ALPS index compared to females. Impaired glymphatic function was linked to both GMV loss and cognitive deficits, with right superior temporal and left postcentral GMV mediating glymphatic-cognitive associations, particularly in executive function and processing speed.

**Conclusion:**

Glymphatic dysfunction in CSVD, particularly in cognitive impairment stage, is closely related to brain atrophy and cognitive decline, supporting the potential utility of glymphatic metrics as clinically imaging biomarkers for assessing cognitive impairment risk and monitor disease progression in CSVD.

## Highlights

• Glymphatic dysfunction was found in CSVD, especially with cognitive impairment.
• Males showed severer glymphatic dysfunction, highlighting sex-specific differences.
• Glymphatic dysfunction was associated with brain atrophy and cognitive function.
• Gray matter atrophy linked glymphatic dysfunction to cognitive impairment in CSVD.

## Graphical abstract

**Figure:**
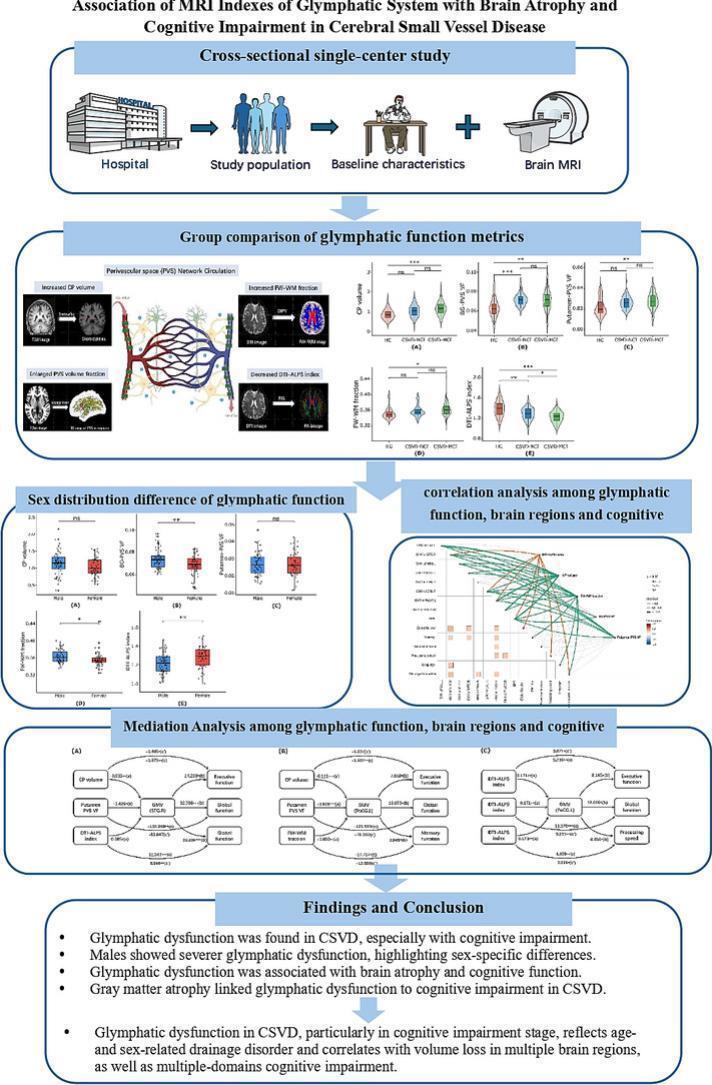

## 1 Introduction

Dementia has emerged as a major public health challenge among the Chinese population, affecting individuals aged 60 years and older. The age- and sex-adjusted prevalence of all-cause dementia in this population is estimated at 6.0%, with vascular dementia (VaD) accounting for 1.6%, making it the second most common form of dementia after Alzheimer’s disease (AD) (Jia et al., 2020). Cerebral small vessel disease (CSVD), the most common vascular disease impacting the brain’s small vessels, is recognized as the primary contributor to vascular cognitive impairment and dementia (Duering et al., 2023, Wardlaw et al., 2019). A range of magnetic resonance imaging (MRI) markers, including white matter hyperintensities (WMHs), lacunes of presumed vascular origin, cerebral microbleeds (CMBs), enlarged perivascular spaces (ePVS), and cerebral atrophy, are recognized as pathological manifestations of CSVD (Duering et al., 2023). However, the underlying mechanisms driving CSVD development, progression, and concomitant cognitive impairment remain poorly understood, with disease-modifying therapies currently lacking. Elucidating the mechanisms may promote new diagnostic and therapeutic strategies to prevent or slow disease advancement.

**Fig. 1:**
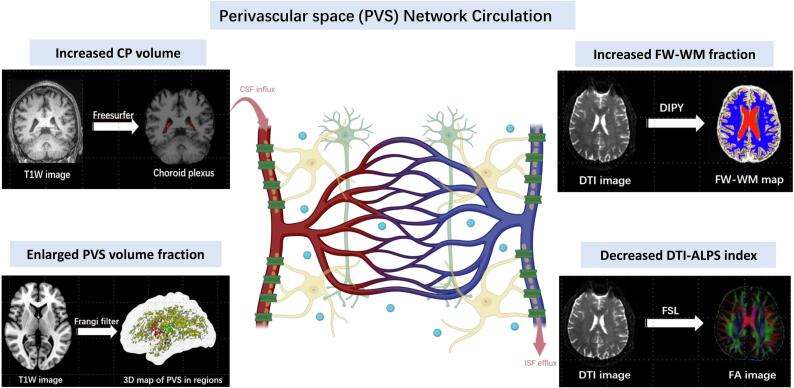
Diagram of MRI indexes of the perivascular space (PVS) network circulation. Cerebrospinal fluid (CSF) production, localized to the choroid plexus, is quantified via choroid plexus volume (CPV) measurements obtained through automated FreeSurfer segmentation. Periarterial CSF influx into the glymphatic pathway is assessed through perivascular space (PVS) volume fraction (PVSVF), calculated using the FreeSurfer pipeline and Frangi filter algorithm. Exchange between periarterial cerebrospinal fluid and parenchymal interstitial fluid (ISF), mediated by aquaporin-4 (AQP4) water channels, is represented by free water fraction in white matter (FW-WM), derived from single-shell diffusion tensor imaging (DTI). Glymphatic clearance efficiency, reflecting perivenous efflux of metabolic waste, is evaluated through the diffusion tensor imaging analysis along the PVS (DTI-ALPS) index, calculated using DTI and ITK-SNAP software. In CSVD pathology, glymphatic dysfunction manifests as: (1) choroid plexus hypertrophy (increased CPV), (2) PVS dilatation (enlarged PVSVF), (3) impaired AQP4-mediated ISF exchange (increased FW-WM fraction), and (4) reduced perivenous clearance capacity (decreased DTI-ALPS index). Abbreviation: DTI-ALPS: diffusion tensor imaging along the perivascular space; FW-WM: free water in white matter; ISF: interstitial fluid; PVS: perivascular space; CSVD: cerebral small vessel disease; CSF: cerebrospinal fluid; CPV: choroid plexus volume. Created with BioRender.com.

The glymphatic system is a recently discovered macroscopic waste clearance system that facilitates waste removal from the central nervous system through cerebrospinal fluid (CSF) transport along perivascular space (Iliff et al., 2012, Klostranec et al., 2021) (Fig. 1). Within the glymphatic system, CSF produced by the choroid plexus (CP) enters the brain parenchyma via *para*-arterial space, driven by aquaporin-4 (AQP4) water channels expressed on astrocytic endfeet. After exchanging with interstitial fluid (ISF), the fluid-metabolite mixture exits through *para*-venous pathways, ultimately directing solutes toward meningeal and cervical lymphatic drainage sites (Mestre et al., 2020). Glymphatic impairment has been proposed as a potential final common pathway in dementia pathogenesis (Nedergaard & Goldman, 2020). While Gadolinium-based contrast agent (GBCA) enhanced MRI remains the gold standard for evaluating glymphatic function *in vivo* (Zhang et al., 2021), its invasive nature has restricted widespread human application. Consequently, non-invasive MRI-based techniques without tracers have emerged as valuable alternatives for indirectly assessing glymphatic activity.

CP, located in the ventricles, serves as a critical regulator of CSF production and has been proposed an indirect imaging biomarker of CSF dynamics and glymphatic function (Hablitz and Nedergaard, 2021). Enlarged CP volume has been consistently associated with reduced CSF turnover rates across multiple aging-related pathologies, such as AD (Choi et al., 2022, Li et al., 2023). MRI-visible perivascular spaces (PVS) are considered to represent periarterial space rather than perivenous space (Wardlaw et al., 2020). Therefore, these spaces are believed to constitute the influx routes of the glymphatic system, facilitating CSF entry into the brain parenchyma. The dilation of PVS is considered a potential structural marker of impaired glymphatic clearance pathways, secondary to ISF drainage obstruction (Mestre et al., 2017, Wardlaw et al., 2013). Free water in white matter (FW-WM) estimates extracellular water volume fraction (Li et al., 2024a), which may be modulated by ISF within the brain parenchyma. Increased FW-WM fraction is considered as a surrogate measure potentially indicative of interstitial fluid retention resulting from glymphatic dysfunction (Andica et al., 2023, Kamagata et al., 2022). Studies in AQP4-deficient mice have demonstrated increased water diffusivity, suggesting expanded extracellular volume and impaired fluid dynamics (Gomolka et al., 2023). The diffusion tensor imaging analysis along perivascular spaces (DTI-ALPS) method, introduced by Taoka et al., quantifies water diffusion along perivascular routes adjacent to deep medullary veins using dynamic contrast-enhanced imaging (Taoka et al., 2017). This technique demonstrates strong correlation with tracer-based measurements of glymphatic clearance and has been considered an indirect alternative indicator reflecting the efficiency of glymphatic clearance along the perivascular space (Zhang et al., 2021).

Brain atrophy represents a hallmark of normal aging, accompanied by heterogeneous pathological alterations. Accumulating evidence links progressive cerebral atrophy to CSVD (Wardlaw et al., 2013), which manifests through distinct patterns of subcortical atrophy, such as ventricular enlargement, and cortical atrophy characterized by sulcal widening and cortical thinning (Jouvent et al., 2020). Although prior studies have linked individual glymphatic metrics with CSVD-related imaging biomarkers and clinical symptoms, such as WMHs, lacunar infarction, and cognitive impairment (Li et al., 2023, Tian et al., 2023), the independent and synergistic effects of multiple glymphatic markers within a unified framework, along with their voxel-wise spatial relationship with gray matter atrophy and their potential indirect effects on domain-specific cognition, remain systematically unexplored. Moreover, it remains unclear whether the observed cognitive deficits are directly attributable to glymphatic dysfunction, or indirectly mediated through its detrimental impact on brain structural integrity.

To address these gaps, this study integrates multi-modal glymphatic imaging measures combined with whole-brain voxel-based morphometry (VBM) to systematically evaluate the relative contributions of different glymphatic indices to gray matter structure and cognitive performance, and further investigates the potential mediating role of key regional gray matter alterations in the relationship between glymphatic function and cognitive outcomes. These analyses not only advance our understanding of the underlying pathophysiology, but may also yield non-invasive imaging biomarkers for future disease monitoring and early detection.

## 2 Materials and methods

### 2.1 Participants

In this study, a total of 120 CSVD participants, including 52 with no cognitive impairment (CSVD-NCI) and 68 with mild cognitive impairment (CSVD-MCI), were included. Inclusion criteria included: (1) age 50–80 years; (2) native Chinese speakers and right-handed; (3) MRI meeting the imaging standards for CSVD diagnosis (Wardlaw et al., 2013), at least meet one of the following aspects; WMH: periventricular WMH (PWMH) Fazekas score = 3 or deep WMH (DWMH) Fazekas score ≥2; CMBs: ≥1; and lacunes: ≥1. Exclusion criteria included: (1) craniocerebral injury; (2) history of tumor; (3) severe cardiovascular, liver, or kidney dysfunction; (4) inability to complete inspections. During the same period, 40 healthy controls (HCs) were recruited. The inclusion criteria for HCs were as follows: (1) age 50–80 years; (2) native Chinese speakers and right-handed; (3) normal neurological examination, absence of subjective cognitive complaints, a MoCA score ≥26, and a global CDR score of 0; (4) MRI demonstrating no significant infarction, hemorrhage, or space-occupying lesions; a Fazekas WMH score ≤1; absence of cerebral microbleeds; and absence of significant large vessel stenosis; (5) no history of neurological or psychiatric disorders (e.g., stroke, dementia, epilepsy, depression); (6) absence of severe or unstable systemic diseases (e.g., significant cardiac, hepatic, or renal insufficiency, or malignancy).

### 2.2 Baseline clinical characteristics and classification of cognitive function

Demographic characteristics, including age, sex, and years of education, were recorded for each subject by a trained neurologist. The vascular risk factors (VRFs) included hypertension, diabetes, hypercholesterolemia, smoking, and body mass index (BMI) using a standardized questionnaire (van Norden et al., 2011, Gottesman et al., 2017).

Patients with CSVD were divided into two groups according to the clinical dementia rating (CDR) score (Gorelick et al., 2011): participants with mild cognitive impairment (CDR = 0.5, CSVD-MCI group) and participants with no cognitive impairment (CDR = 0, CSVD-NCI group).

### 2.3 Neuropsychological assessment

All participants underwent a neuropsychological assessment in a quiet, comfortable environment within a week of their MRI examination, conducted by an experienced neurologist. Chinese Montreal Cognitive Assessment (MoCA) were employed to assessed the global cognitive function. Specific cognitive domains were assessed with: memory function (Auditory Verbal Learning Test (AVLT)), executive function (Trail Making Test-B (TMT-B)), processing speed (Trail Making Test-A (TMT-A)), visuospatial function (10-point clock drawing test (CDT-10)), and language function (Boston Naming Test (BNT)). The z-transformation were calculated for each cognitive domain (z-score = individual test score minus means of healthy controls, divided by standard deviation of controls) to compare cognitive function between groups (Wang et al., 2023). Notably, the original scores of the TMT were based on the completion time; therefore, the z-scores of the processing speed and executive function were inverted by multiplying them by −1, with higher z-scores representing better performance (Huang et al., 2020).

### 2.4 MRI protocols

MRI data were obtained using a 3.0-Tesla MR system (Discovery MR750w, General Electric, Milwaukee, WI, USA) with a 24-channel head coil (Supplementary eMethod for details).

### 2.5 Quantification of brain parenchymal fraction (BPF)

Based on high-resolution three-dimensional T1-weighted (3D-T1) images, FreeSurfer version 6.0 (https://surfer.nmr.mgh.harvard.edu/) was used for data processing and brain tissue segmentation to calculate the brain parenchymal fraction (BPF), as previously described (Fischl, 2012). In brief, pre-processing included image bias field correction, skull stripping to remove non-brain structures, identification of gray and white matter boundaries in each hemisphere of the cortex, and segmentation of brain tissue into white matter (WM), gray matter (GM), and CSF. All the final automatic segmentations were visually inspected for accuracy by one neuroimaging researcher. Total intracranial volume (TIV) was the sum of the volumes of GM, WM, and CSF. The brain parenchymal fraction (BPF) was defined as the ratio of brain tissue volume (GM and WM volume) to the TIV.

### 2.6 Choroid plexus volume (CPV) segmentation

Based on high-resolution three-dimensional T1-weighted (3D-T1) images, automatic segmentation was performed using FreeSurfer version 6.0 to quantify choroid plexus volume (CPV). The volume of both left and right choroid plexus was individually measured and then summed to derive the total CPV for each participant. To reduce inter-participant variability, the segmented CP volume was expressed as the ratio of CP volume to TIV multiplied by 1000, as previously described (Choi et al., 2022). Therefore, CPV/TIV ratio (*1000) will be referred in this paper as CP volume.

### 2.7 PVS mapping

PVS mapping was performed using an Enhanced PVS Contrast (EPC) method applied to both T1-weighted and T2-weighted images with removing non-structured high frequency spatial noise through a dedicated filtering algorithm (Sepehrband et al., 2019). After preprocessing (intensity normalization, skull stripping), FLAIR images were corrected for inhomogeneity and co-registered to Montreal Neurological Institute (MNI)-152 space. Subsequently, white matter and basal ganglia (including subfields: amygdala, pallidum, caudate, and putamen) were segmented using FMRIB Software Library V.6.0 (FSL, https://www.fmrib.ox.ac.uk/fsl/). Last, filtered T1w/T2w ratios generated EPC images, followed by Frangi filtering to quantify vesselness. The PVS volume fraction was calculated as the ratio of PVS volume to the sum of gray matter and white matter volumes, eliminating effects of interindividual brain size variability. The equation for PVS volume faction is as follows:

$$\mathrm{PVS}\;volume\;fraction\;\left(PVS\;VF\right)=\frac{\;PVS\;volume}{\mathrm{gray}+\;white\;matter\;volume}\times 100\%$$

### 2.8 Quantification of DTI-ALPS index

The calculation of the DTI-ALPS index is based on the diffusion tensor imaging (DTI) sequence, and the processing flow is as follows: DTI data were processed using FSL version 6.0, including non-brain tissues removal, head motion, eddy current correction, and DTI parameter calculation (fractional anisotropy [FA] and color-coded FA map). Two 5-mm diameter spherical regions of interest (ROIs) were placed on the same x-axis in projection and the association fibers areas, where the deep medullary veins were vertical to the ventricle body. Diffusivities in the directions of the x-axis (Dxx), y-axis (Dyy), and z-axis (Dzz) of each ROI were recorded. The DTI-ALPS index was calculated only in the left hemisphere because all participants were right-handed, making the left hemisphere dominant with more developed association fibers relevant to cognition and thus a more representative measurement site. Furthermore, unilateral assessment is a common practice in the DTI-ALPS field, which helps avoid confounding factors introduced by inter-hemispheric variability, ensures methodological consistency, and aligns with established protocols (Taoka et al., 2017, Tian et al., 2023). The equation for DTI-ALPS index is as follows:

$$\mathrm{DTI}-ALPS\;index\;=\frac{mean\left(Dxxproj,Dxxassoc\right)}{mean\left(Dyyproj\;,Dzzassoc\right)}$$

### 2.9 Calculation of FW-WM fraction

The calculation of the FW-WM fraction is based on the DTI sequence and is carried out using the single-shell free water estimation model in the DIPY (version 1.4.0) package in Python (https://nipy. org/dipy/index.html) (Pasternak et al., 2009). Briefly, the signal was fitted to a two-compartment model in each voxel, including a FW compartment (isotropic tensor) and a tissue compartment (FW-corrected tensor). The FW map represents the relative fraction of FW in each voxel, ranging from 0 to 1. Finally, the FW fraction across a mean FA skeleton of the whole-brain WM were obtained.

### 2.10 Voxel-based morphometry (VBM) analysis

VBM analysis was performed using the Computational Anatomy Toolbox 12 (CAT12) within SPM12. Derived from the initial tissue segmentation as described in Section 2.5, a high-dimensional DARTEL registration was used to create a study-specific template and to normalize the GM images to the Montreal Neurological Institute (MNI) space. The normalized images were modulated to preserve the total amount of tissue volume and were subsequently smoothed with an 8-mm full-width at half-maximum Gaussian kernel (FWHM). Family-wise error (FWE) correction at the cluster level addressed multiple comparisons, setting the criterion for statistical significance at P < 0.05, adjusted to P = 0.001 at the voxel level (Eklund et al., 2016). Data processing and analysis for brain imaging (DPABI) software (https://rfmri.org/dpabi) was used to extract GMV values of specific structurally changed brain regions for region-of-interest analysis (Yan et al., 2016).

### 2.11 Statistical analysis

Statistical analysis was performed using R software (version 4.1.1) and IBM SPSS Statistics (version 26). Normally distributed continuous variables were expressed as mean ± standard deviation (Mean ± SD) and compared using one-way analysis of variance (ANOVA), while non-normally distributed variables were expressed as median (interquartile range, IQR) and analyzed using Kruskal-Wallis test. Categorical variables were presented as counts (percentages, %) and assessed with chi-square test. Group comparisons of glymphatic function metrics were performed with Bonferroni correction, controlling for age, sex, and VRFs (corrected *P* < 0.05). SPM software was utilized to assess correlations between glymphatic function and whole-brain GMV, controlling for age, sex, education, and TIV, with FWE correction (corrected *P* < 0.05). Partial correlations analysis between glymphatic function metrics and brain GMV controlled for demographics, VRFs, and TIV. Partial correlations analysis of glymphatic function metrics and cognition adjusted for demographics, VRFs, and neuroimaging markers of CSVD. Similarly, partial correlation analysis relating brain GMV to cognition and mediation analysis included the full set of demographics, VRFs, neuroimaging markers, and TIV covariates. For correlation analyses between glymphatic function metrics, brain GMV and cognition, false discovery rate (FDR) correction was applied, with a significance threshold set at *P_FDR_* < 0.05. PROCESS macro (https://www. processmacro.org/) software was used for mediation analysis. Based on 5000 bootstrap realizations, a significant indirect effect was determined if the bootstrap 95% confidence interval (CI) excluded zero. For mediation analyses, given the complexity of path modeling and the fact that bootstrap confidence intervals inherently provide a test of indirect effects, no further multiple comparisons correction was applied. Statistical significance was set at *P* < 0.05.

## 3 Results

### 3.1 Demographic and clinical characteristics

As summarized in Table 1, patients in CSVD-MCI group were older than HC group, with both the CSVD subgroups having higher proportion of hypertension. No significant intergroup differences were observed in sex distribution, education level, diabetes, hypercholesterolemia, smoking status, or body mass index. Cognitive performance was markedly impaired in the CSVD-MCI group compared with both the CSVD-NCI and HC groups. Both CSVD subgroups exhibited more severe WMHs, a greater burden of lacunes and CMBs, and reduced BPF compared with HCs. Furthermore, the CSVD-MCI group demonstrated significantly higher WMHs than the CSVD-NCI group.

**Table 1:** Baseline Demographic, Cognitive Function, and Neuroimaging Characteristics among HC, CSVD-NCI, and CSVD-MCI groups.

	HC (n = 40)	CSVD-NCI (n = 52)	CSVD-MCI (n = 68)	F/λ-value	*P*-value	

Demographics						
Age, years	60.18 ± 4.57	62.38 ± 6.96	64.69 ± 6.62 #	6.6624	**0.0017**^a^	
Male, n (%)	19 (47.50)	28 (53.85)	33 (48.53)	0.4665	0.7919^b^	
Education, years	9.43 ± 4.10	9.09 ± 3.85	8.10 ± 3.80	1.7355	0.1797 a	
Smoking, n (%)	5 (12.50)	16 (30.77)	21 (30.88)	5.2087	0.0740^b^	
Hypertension, n (%)	14 (35.00)	33 (63.46)†	45 (66.18) #	11.1375	**0.0038**^b^	
Diabetes, n (%)	6 (15.00)	9 (17.31)	12 (17.65)	0.1361	0.9342^b^	
Hypercholesterolemia, n (%)	10 (25.00)	15 (28.85)	19 (27.94)	0.1793	0.9143^b^	
BMI (kg/m^2^)	23.77 ± 2.28	23.78 ± 2.55	23.89 ± 3.02	0.0351	0.9655 a	
Neuropsychological assessments						
MoCA total scores	25.98 ± 1.86	25.25 ± 2.29	19.82 ± 3.85 #, §	73.1201	**<0.001**^a^	
CDR scores	0.00 ± 0.00	0.00 ± 0.00	0.49 ± 0.09 #, §	1489.5375	**<0.001**^a^	
Memory function	0.00 ± 1.00	−0.21 ± 1.01	−1.16 ± 1.46 #, §	14.3844	**<0.001**^a^	
Executive function	0.00 ± 1.00	−0.59 ± 1.92	−2.33 ± 2.87 #, §	16.4734	**<0.001**^a^	
Processing speed	0.00 ± 1.00	−0.34 ± 1.39	−1.54 ± 2.45 #, §	10.7523	**<0.001**^a^	
Language function	0.00 ± 2.92	−0.09 ± 3.26	−2.37 ± 3.41 #, §	9.9954	**<0.001**^a^	
Visuospatial function	0.00 ± 1.00	−0.29 ± 1.14	−4.19 ± 4.69 #, §	31.5246	**<0.001**^a^	
Neuroimaging measurements						
WMH Fazekas scores	1.00(0.00, 2.00)	4.00(3.00, 5.00)†	5.00(4.00, 6.00) #, §	104.1848	**<0.001**^c^	
Lacunes, n (%)	0 (0.00)	14 (26.92)†	16 (23.53) #	12.5305	**0.0019**^b^	
CMBs, n (%)	0 (0.00)	22 (42.31)†	33 (48.53) #	28.4421	**<0.001**^b^	
BPF	0.76 ± 0.03	0.74 ± 0.04†	0.73 ± 0.05#	10.47	**<0.001**^a^	

Note: Data are given as n (%) and mean (SD). Each cognitive domain is represented as Z scores.

Abbreviations: HC, healthy control; CSVD, cerebral small vessel disease; NCI, no cognitive impairment; MCI, mild cognitive impairment; BMI, body mass index; MoCA, Montreal Cognitive Assessment; CDR, Clinical Dementia Rating; WMH, white matter hyperintensity; CMBs, cerebral microbleeds; BPF, brain parenchymal fraction.

a One-way analysis of variance (ANOVA).

b Chi-square test.

c Kruskal-Wallis rank-sum test.

† Significant difference between the CSVD-NCI group compared to the HC group (Bonferroni correction, p < 0.05).

# Significant difference between the CSVD-MCI group compared to the HC group (Bonferroni correction, p < 0.05).

§ Significant difference between the CSVD-MCI group compared to the CSVD-NCI group (Bonferroni correction, p < 0.05).

### 3.2 Group differences in glymphatic function metrics

**Fig. 2:**
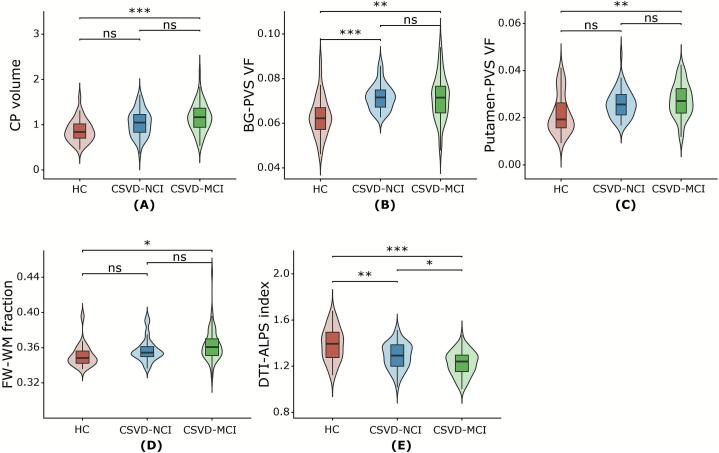
Difference of glymphatic function metrics in HC, CSVD-NCI, and CSVD-MCI groups, corrected for age, sex, and VRFs (hypertension, diabetes, hypercholesterolemia, smoking, and BMI) (Bonferroni correction, p < 0.05). Abbreviation: HC, healthy control; CSVD, cerebral small vessel disease; NCI, no cognitive impairment; MCI, mild cognitive impairment; CP, choroid plexus; BG, basal ganglia; PVS VF, perivascular space volume fraction; FW-WM, white matter free water; DTI-ALPS, diffusion tensor imaging analysis along the perivascular space, VRFs, vascular risk factors; BMI, body mass index. ns not statistically significant; *P < 0.05, **P < 0.01, ***P < 0.001.

We first conducted correlation analyses between whole-brain PVS volume and glymphatic metrics (Supplementary Fig. S1) to identify the most stable and consistently associated PVS regions related to glymphatic dysfunction. Based on two a priori criteria, multi-metric consistency and literature support, we selected the basal ganglia and putamen as core regions for analysis. Specifically, PVS volumes in these regions showed significant associations with multiple glymphatic indicators and a hold well-established biological relevance in CSVD pathophysiology. After controlling for age, sex, and VRFs, the CSVD-MCI group exhibited increased CP volume, FW-WM fraction, BG-PVS, and putamen-PVS volume, alongside a reduced DTI-ALPS index relative to HCs (all *P* < 0.05). A similar pattern of alterations was observed in BG-PVS and DTI-ALPS index when comparing CSVD-NCI and HC groups (all *P* < 0.01). Furthermore, CSVD subgroups analysis exhibited lower DTI-ALPS index in the CSVD-MCI group (all *P* < 0.05) (Fig. 2 and Supplementary Table S3).

### 3.3 Group differences in brain GMV

**Fig. 3:**
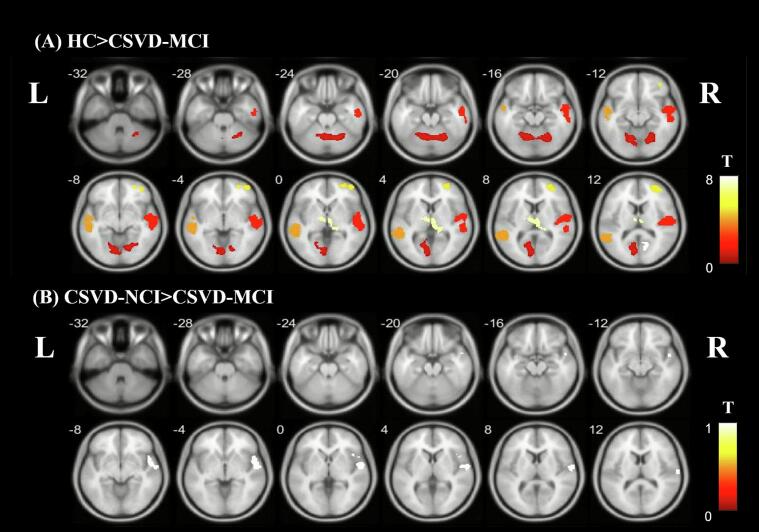
Whole brain group differences in GMV. (A) Differences of brain GMV between HC and CSVD-MCI, (B) Differences of brain GMV between CSVD-NCI and CSVD-MCI, (The color bar represents the T-value from intergroup comparisons, FWE cluster-level correction, *P* < 0.05). Abbreviation: GMV, gray matter volume; HC, healthy control; CSVD, cerebral small vessel disease; NCI, no cognitive impairment; MCI, mild cognitive impairment; FWE, family-wise error. L, left; R, right.

Compared to HCs, patients with CSVD-MCI exhibited significant gray matter atrophy across multiple regions, including the bilateral calcarine fissure, left middle temporal and postcentral gyri, right superior temporal, middle frontal, and precentral gyri, along with the right thalamus (Fig. 3A and Supplementary Table S4). In contrast, comparison between CSVD subgroups revealed more localized atrophy, primarily within the right superior temporal gyrus (Fig. 3B and Supplementary Table S4).

### 3.4 Associations between glymphatic function metrics and demographic and VRFs in CSVD group

**Fig. 4:**
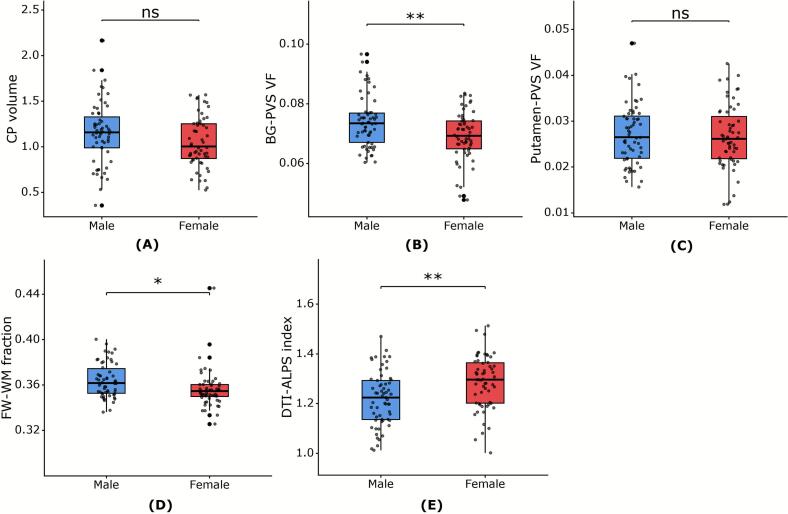
Comparison of sex distribution difference in glymphatic function metrics among CSVD patients, with controlled for age and VRFs (hypertension, diabetes, hypercholesterolemia, smoking, and BMI). Abbreviations: CSVD, cerebral small vessel disease; CP, choroid plexus; BG, basal ganglia; PVS VF, perivascular space volume fraction; FW-WM, white matter free water; DTI-ALPS, diffusion tensor imaging analysis along the perivascular space; VRFs, vascular risk factors; BMI, body mass index. ns not statistically significant, *P < 0.05, **P < 0.01, ***P < 0.001.

To identify potential determinants of glymphatic function, we examined partial correlations between glymphatic function metrics and key demographic and vascular risk factors within the CSVD group. Within the CSVD group, older age was associated with larger CP volume (*P* = 0.002), increased PVS volume in the BG (*P* = 0.028) and putamen (*P* = 0.022), otherwise lower DTI-ALPS index (*P* = 0.018). After controlling for age and VRFs, a significant main effect of sex was observed in the BG-PVS, FW-WM fraction and DTI-ALPS index, where males demonstrated significantly larger BG-PVS VF and FW-WM fraction, as well as lower DTI-ALPS index compared to females (all *P* <  0.05) (Fig. 4 and Supplementary Table S5).

To quantify the magnitude of sex differences, we calculated Cohen’s d with 95% confidence intervals (CIs) after controlling age and VRFs. We observed a medium effect size for DTI-ALPS index (Cohen’s d = −0.59, 95% CI: [-0.96, −0.22], p = 0.002), indicating that males had substantially lower DTI-ALPS index than females. The effect for BG-PVS VF (Cohen’s d = 0.49, 95% CI: [0.13, 0.86], p = 0.009) and FW-WM fraction (Cohen’s d = 0.46, 95% CI: [0.09, 0.83], p = 0.017) were small (Supplementary Table S6). In our analysis of interaction effects, specifically between sex and diagnostic group, and between sex and age, on glymphatic metrics, none of the interaction terms reached formal statistical significance in either model (all *P* > 0.05) (Supplementary Table S7 and S8).

To examine whether the observed sex differences in glymphatic function were accompanied by corresponding differences in brain structure or cognition, supplemental analyses were conducted within the CSVD patient group. After adjusting for age, VRFs, and TIV, no statistically significant sex differences were observed in the GMV of key regions of interest or in the BPF (all *P* > 0.05) (Supplementary Fig. S2). Similarly, after controlling for age, VRFs, and CSVD neuroimaging markers, no significant sex differences were detected in global cognitive performance or in any domain–specific cognitive performance (all *P* > 0.05) (Supplementary Fig. S3).

### 3.5 Correlation analysis among glymphatic function metrics, brain GMV, and cognitive function in CSVD group

**Fig. 5:**
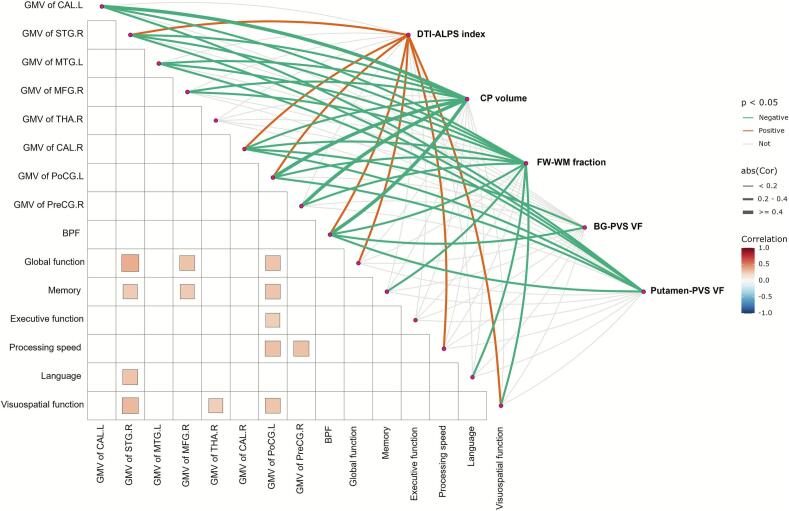
The relationship between glymphatic function metrics, GMV of brain regions and cognitive function in CSVD group. Color intensity represents the magnitude of partial correlation coefficients (r) with red indicating positive correlations and blue indicating negative correlations. Only statistically significant correlations after FDR correction (*P* < 0.05) are displayed. Abbreviation: CSVD, cerebral small vessel disease; DTI-ALPS, diffusion tensor imaging analysis along the perivascular space; CP, choroid plexus; FW-WM, white matter free water; BG, basal ganglia; PVS VF, perivascular space volume fraction; GMV, gray matter volume; CAL.L, left calcarine fissure; CAL.R, right calcarine fissure; MTG.L, left middle temporal gyrus; MFG.R, right middle frontal gyrus; PoCG.L, left postcentral gyrus; PreCG.R, right precentral gyrus; STG.R, right superior temporal gyrus; THA.R, right thalamus; BPF, brain parenchymal fraction; FDR, false discovery rate. (For interpretation of the references to colour in this figure legend, the reader is referred to the web version of this article.)

As show in Fig. 5 and Supplementary Table S9, CP volume negatively correlated with GMV in bilateral temporal regions, the calcarine fissure, along with postcentral gyrus in CSVD group (all *P* < 0.05). Putamen-PVS volume exhibited negative correlations with GMV in the left (r = −0.229, *P* = 0.015) and right calcarine fissure (r = −0.369, *P* < 0.001), right superior temporal gyrus (r = −0.280, *P* = 0.003), left middle temporal gyrus (r = −0.288, *P* = 0.002), and left postcentral gyrus (r = −0.361, *P* < 0.001), while DTI-ALPS index positively linked to GMV of left postcentral gyrus (r = 0.282, *P* = 0.003), right superior temporal gyrus (r = 0.351, *P* < 0.001) and calcarine fissure (r = 0.328, *P* < 0.001). All glymphatic function metrics are closely related to BPF (all P < 0.01).

In CSVD group, the DTI-ALPS index demonstrated positive correlations with global cognition (r = 0.328, *P* < 0.001), processing speed (r = 0.232, *P* = 0.015) and visuospatial function (r = 0.286, *P* = 0.003), while FW-WM fraction was inversely associated with memory (r = −0.265, *P* = 0.007), language (r = −0.298, *P* = 0.002), and visuospatial function (r = −0.284, *P* = 0.004) (Fig. 5 and Supplementary Table S10). Regional GMV analyses showed the GMV of right superior temporal gyrus was positively associated with global cognitive (r = 0.353, *P* < 0.001) and memory functions (r = 0.252, *P* = 0.009), GMV of right middle frontal gyrus positively similarly contributed to global cognitive (r = 0.276, *P* = 0.004) and memory functions (r = 0.251, *P* = 0.009), while the GMV of left postcentral gyrus exhibited the most extensive involvement, associating with executive function (r = 0.240, *P* = 0.012), processing speed (r = 0.284, *P* = 0.003), memory (r = 0.270, *P* = 0.005), and visuospatial functions (r = 0.273, *P* = 0.004) (Fig. 5 and Supplementary Table S11).

### 3.6 Mediation analysis among glymphatic function metrics, brain GMV, and cognitive function in CSVD group

**Fig. 6:**
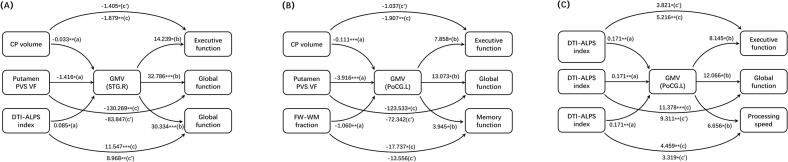
Brain regions mediate the relationship of glymphatic function to cognitive function metrics in CSVD group, corrected for demographics, VRFs, neuroimaging makers, and TIV. In the mediation model, path c represents the total effect of the independent variable (X, glymphatic metric) on the dependent variable (Y, cognitive function). Path c′ indicates the direct effect of X on Y after controlling for the mediator (M, regional GMV). Path a indicates the effect of X on M, while path b represents the effect of M on Y after controlling for X. The indirect effect is estimated by the product term a × b, representing the effect of the independent variable on the dependent variable through the mediator, with its significance tested via bootstrap confidence intervals. Path coefficients with P values (*P < 0.05, ** *P* < 0.01, *** *P* < 0.001, respectively). Abbreviation: CSVD, cerebral small vessel disease; CP, choroid plexus; PVS VF, perivascular space volume fraction; FW-WM, white matter free water; DTI-ALPS, diffusion tensor imaging analysis along the perivascular space; GMV, gray matter volume; PoCG.L, left postcentral gyrus; STG.R, right superior temporal gyrus; VRFs, vascular risk factors; CI: confidence interval; TIV: total intracranial volume.

In CSVD group, mediation analysis revealed that the GMV of right superior temporal gyrus mediate the relationship between CP volume and executive function (mediation effect% = 25.2%, indirect effect: −0.474, 95% CI [-1.021, −0.031]), putamen-PVS volume and global function (mediation effect% = 35.6%, indirect effect: −46.422, 95% CI [-94.593, −7.749]), as well as DTI-ALPS index and global function (mediation effect% = 22.3%, indirect effect: 2.579, 95% CI [0.350, 5.722]) (Fig. 6A). The left postcentral gyrus emerged as a more extensive mediator, connecting CP volume to executive function (mediation effect% = 45.6%, indirect effect: −0.870, 95% CI [-1.741, −0.081]), putamen PVS volume to global function (mediation effect% = 41.4%, indirect effect: −51.191, 95% CI [-98.375, −9.319]), and FW-WM fraction to memory (mediation effect% = 23.6%, indirect effect: −4.181, 95% CI [-14.106, −0.761]) (Fig. 6B). The GMV of left postcentral gyrus mediated relationships between DTI-ALPS index and multiple cognition domains, including global cognition (mediation effect% =18.2%, indirect effect: 2.067, 95% CI [0.246, 4.611]), executive function (mediation effect% = 26.8%, indirect effect: 1.395, 95% CI [0.119, 3.222]), and processing speed (mediation effect% = 25.6%, indirect effect: 1.140, 95% CI [0.014, 2.815]) (Fig. 6C).

## 4 Discussion

Our study demonstrates that glymphatic dysfunction, assessed through multimodal MRI indices, is significantly associated with gray matter atrophy and cognitive impairment in CSVD. Specifically, we identified three key findings: (1) patients with CSVD-MCI exhibit pronounced alterations across multiple glymphatic metrics, including increased CP volume, enlarged PVS volume in the BG and putamen, elevated FW-WM fraction, along with reduced DTI-ALPS index; (2) sex-specific differences in glymphatic metrics were identified, with male patients demonstrating larger BG-PVS volume, higher FW-WM fraction, and lower DTI-ALPS index compared to female; and (3) gray matter volume loss, particularly in temporal and postcentral regions, partially mediates the relationship between glymphatic impairment and cognitive decline, especially in domains of executive function and processing speed.

The pattern of glymphatic alterations observed in CSVD-MCI suggests a state of impaired fluid influx, interstitial stasis, and compromised clearance efficiency. The observed enlargement of the choroid plexus, a structure central to CSF production, has been linked to impaired fluid exchange and neuroinflammatory activation (Choi et al., 2022, Althubaity et al., 2022). Concurrently, the dilation of PVS in the BG likely reflects structural remodeling of the periarterial influx routes, possibly secondary to impaired interstitial fluid drainage (Mestre et al., 2017, Wardlaw et al., 2020). The elevation in FW-WM fraction further indicates dysregulation of parenchymal fluid homeostasis, potentially attributable to inefficient aquaporin-4–mediated fluid exchange at the astrocytic endfeet (Huang et al., 2022, Planetta et al., 2016, Sacchi et al., 2024). Finally, the reduction in the DTI-ALPS index, a putative marker of perivascular clearance efficiency, reinforces the notion of impaired glymphatic efflux (Taoka et al., 2017, Zhang et al., 2021). Notably, BG-PVS enlargement and DTI-ALPS reduction were already detectable in cognitively normal CSVD patients, indicating early perivascular dysfunction, whereas choroid plexus hypertrophy and free water accumulation emerged predominantly in the MCI stage. This temporal evolution implies a shift from localized perivascular impairment to a more systemic glymphatic failure as CSVD advances, potentially accelerating cognitive decline.

Consistent with prior findings (Alisch et al., 2021), glymphatic function exhibits age-dependent impairment. A secondary yet notable finding was the more pronounced glymphatic dysfunction observed in male patients across several metrics, aligning with some prior observations (Zhang et al., 2021, Choi et al., 2022, Tian et al., 2023). However, supplemental analyses within the CSVD cohort revealed no corresponding sex differences in regional gray matter volume or cognitive performance after controlling for age, vascular risk factors, and disease burden. This dissociation suggests that glymphatic dysfunction may represent a relatively early or sensitive event in the CSVD pathophysiological cascade, possibly preceding detectable divergence in macrostructural brain integrity or overt cognitive performance between sexes. The mechanisms underlying this sex-specific vulnerability remain speculative but could involve hormonal modulation of neuroinflammatory tone, blood–brain barrier integrity, or vascular reactivity. For instance, preclinical evidence suggests that androgens may promote neuroinflammation and vascular vulnerability, whereas estrogens might enhance barrier function (Zuloaga et al., 2012). Nevertheless, in the absence of direct hormonal measures, such interpretations remain hypothetical. We therefore emphasize that these sex-related patterns should be interpreted as a preliminary signal requiring validation in large-scale, multi-center longitudinal cohorts that integrate serum hormone assays, receptor genetics, and multimodal neuroimaging.

Patients with CSVD-MCI exhibited pronounced gray matter atrophy, predominantly in frontal and temporal regions. These areas constitute core nodes within networks that support executive control, sensory integration, and memory processing, which are consistently impaired in CSVD (Lee et al., 2019). This atrophy correlated with both glymphatic dysfunction and cognitive deficits, supporting an interplay between impaired clearance and structural damage. These findings align with previous reports associating CP enlargement with atrophy in cognitively relevant regions (Jiang et al., 2024) and observations in AD linking greater atrophy to lower DTI-ALPS index (Sacchi et al., 2024, Li et al., 2022, Li et al., 2024b). Significant correlations were also observed between multiple glymphatic metrics and cognitive performance, especially in executive function and processing speed, even after controlling for conventional neuroimaging markers of CSVD (Čarna et al., 2023, Edde et al., 2020, Schroeter, 2022). Notably, PVS burden in the putamen, a key node within dorsal striatal circuits that integrates frontal and thalamic inputs to support cognitive-motor functions, was particularly associated with cognitive impairment (Parent and Hazrati, 1995). This aligns with prior reports linking putamen atrophy to cognitive decline in neurodegenerative conditions, underscoring its potential role as a critical hub within the basal ganglia-perivascular network contributing to cognitive dysfunction (de Jong et al., 2008, Cho et al., 2014). Mediation analysis further indicated that gray matter volume in the right superior temporal gyrus and left postcentral gyrus significantly mediated the relationship between glymphatic dysfunction and cognitive decline. The right superior temporal gyrus is a hub for sensory integration and default–mode network function, and its atrophy is linked to deficits in processing speed and executive function. The left postcentral gyrus, while primarily somatosensory, also supports sensory–motor integration and spatial attention through connections with prefrontal and parietal regions. Atrophy in these areas likely disrupts the neural circuits critical for higher–order cognition, helping to explain their mediating role.

Building on these observations, we propose a mechanistic cascade whereby glymphatic failure leads to the accumulation of neurotoxic waste products (e.g., amyloid-β, tau), particularly in metabolically active gray matter regions, which in turn triggers sustained neuroinflammation, disrupts neurovascular coupling, and promotes chronic hypoperfusion and oxidative stress (Klistorner et al., 2022). Together, waste accumulation and sustained neuroinflammation likely disrupt neurovascular unit function, compromising blood–brain barrier integrity and cerebrovascular autoregulation. This cascade may lead to chronic local hypoperfusion and a toxic, energy–deprived microenvironment that ultimately drives neuronal apoptosis. Collectively, these processes manifest as gray matter atrophy and cortical thinning, culminating in dementia (Nedergaard and Goldman, 2020, Tarasoff-Conway et al., 2015). While this cascade offers a plausible mechanism linking microvascular pathology to parenchymal damage in CSVD, our cross‑sectional data cannot establish causality. Alternative pathways, such as primary vascular injury leading to both glymphatic impairment and atrophy, or bidirectional interactions, remain plausible. Notablely, the mediation analyses were exploratory and were not corrected for the full range of potential pathway configurations. The wide confidence intervals observed for several indirect effects indicate limited precision in estimating their magnitude, reflecting constraints in sample size, measurement variability, and potential effect heterogeneity. Therefore, while the presence of a mediating effect is supported, the precise strength of these pathways should be interpreted cautiously. These findings provide preliminary statistical evidence for a glymphatic–structure–cognition relationship rather than definitive mechanistic proof. Future longitudinal and multimodal studies with larger samples are needed to validate this pathway and clarify the directional relationships among glymphatic function, brain structure, and cognition.

From a translational perspective, our findings highlight the potential of multimodal glymphatic imaging, particularly the DTI-ALPS index and BG PVS volume, as sensitive biomarkers for detecting early functional disturbances in CSVD. Unlike conventional structural markers (e.g., WMHs, lacunes), which often reflect accumulated end-stage injury, glymphatic metrics may capture dynamic, pre-structural alterations in brain fluid homeostasis. Integrating these metrics could therefore improve risk stratification, helping to identify individuals with “normal-appearing” structural scans but heightened susceptibility to cognitive decline. Furthermore, the identification of specific brain regions where atrophy mediates the glymphatic-cognitive relationship offers potential targets for future mechanistic investigations and therapeutic strategies aimed at preserving neurovascular and clearance functions.

A key strength of this study lies in the systematic integration of multiple complementary MRI proxies of glymphatic function, providing a more comprehensive assessment than single-metric approaches. The use of region-specific PVS quantification and voxel-wise morphometry further strengthens the anatomical specificity of our findings. However, several limitations should be acknowledged. First, the single-center design and moderate sample size, drawn exclusively from a Han Chinese population, may limit generalizability and statistical power for detecting subtle effects. Given that genetic background and environmental factors can influence disease risk, neuroimaging phenotypes, and glymphatic function, the observed relationships among glymphatic metrics, brain structure, and cognition require validation in larger, multi‑center, and ethnically diverse prospective studies. Second, the cross-sectional design precludes causal inferences regarding the observed relationships. Although we propose a plausible explanatory model based on existing evidence and our statistical findings, the precise causal and temporal sequence linking these variables require validation through longitudinal or interventional studies. Third, sleep architecture, a key modulator of glymphatic activity, was not assessed, representing a potential confounder. Glymphatic clearance is known to be enhanced during slow–wave sleep, and sleep disturbances can impair clearance efficiency, potentially contributing to pathological accumulation and cognitive decline (Fultz et al., 2019, Hablitz and Nedergaard, 2021, Rasmussen et al., 2022, Taoka et al., 2024). Future studies incorporating polysomnography or validated sleep questionnaires are needed to clarify the independent and interactive contributions of sleep to glymphatic function and cognitive decline in CSVD. Fourth, although we adjusted for key vascular risk factors, residual confounding from unmeasured lifestyle or genetic factors cannot be excluded. Future studies would benefit from more rigorous matching or stratification of vascular risk profiles at the recruitment stage to reduce potential systemic bias. Fifth, calculating the DTI-ALPS index only in the left hemisphere, while methodologically consistent with prior studies, may overlook functionally relevant hemispheric asymmetries in glymphatic efficiency. Future studies should systematically evaluate bilateral DTI-ALPS index and further examine whether hemispheric asymmetry ratios correlate specifically with CSVD severity and cognitive profiles for more precise disease characterization. Furthermore, methodological and regional constraints should be noted. PVS mapping relied on non-isotropic, limited–resolution acquisitions, which may have reduced sensitivity to smaller PVS and introduced partial–volume effects. In addition, focusing on the BG and putamen enhanced specificity but may have overlooked potential pathophysiologically relevant PVS in other brain regions. Future studies employing high–resolution, isotropic 3D sequences with whole–brain exploratory analyses will enable more comprehensive quantification and help verify the generalizability of the observed associations. Finally, all glymphatic function metrics employed represent indirect imaging proxies rather than direct measurements. Their correlation with true clearance kinetics in humans remains to be validated against direct intrathecal tracer studies. Although the observed associations with brain atrophy and cognitive impairment supports the plausibility of our proposed framework, these findings should be interpreted as a heuristic model requiring validation through more direct and specific functional assessment techniques.

## 5 Conclusion

In conclusion, this study provides cross-sectional evidence that glymphatic dysfunction in CSVD is associated with regional gray matter atrophy and cognitive impairment, with specific brain regions acting as statistical mediators of this relationship. The multimodal glymphatic metrics employed show potential as functional biomarkers for early detection and progression monitoring. However, considering the methodological limitations, the results should be interpreted with caution and further longitudinal studies are required to validate these pathways, clarify causal mechanisms, and explore their therapeutic relevance.

## Ethical approval and consent to participate

The Institutional Ethics Committee of the First Affiliated Hospital of Anhui Medical University approved this study, which was conducted in accordance with the principles of the Declaration of Helsinki (approval number: PJ2023-01-45). All the subjects were informed and have signed a written consent to participate in this study.

## Funding sources

This work was supported by 10.13039/501100001809National Natural Science Foundation of China (82401420), Key Research and Development Projects of Anhui Province (202104j07020031), 10.13039/501100003995Natural Science Foundation of Anhui Province (2108085MH274), Anhui University Scientific Research Major Project (2022AH040159), the Traditional Chinese Medicine Inheritance and Innovation Research Project of Anhui Province (2024CCCX230), and the Hefei City Health Commission applied medicine project (Hwk2023yb003).

## Declaration of generative AI and AI-assisted technologies in the writing process

Not applicable.

## CRediT authorship contribution statement

**Lulu Ai:** Writing – original draft, Software, Methodology, Investigation, Formal analysis, Data curation, Conceptualization. **Zhiwei Li:** Writing – review & editing, Software, Methodology, Formal analysis, Data curation, Conceptualization. **Chaojuan Huang:** Writing – review & editing, Methodology, Investigation, Data curation, Conceptualization. **Xia Zhou:** Writing – review & editing, Project administration, Funding acquisition, Formal analysis, Data curation. **Xiaoqun Zhu:** Writing – review & editing, Supervision, Resources, Project administration, Funding acquisition, Data curation. **Qiaoqiao Xu:** Writing – review & editing, Supervision, Resources, Project administration, Funding acquisition, Data curation. **Zhongwu Sun:** Writing – review & editing, Supervision, Resources, Project administration, Funding acquisition, Data curation.

## Declaration of competing interest

The authors declare that they have no known competing financial interests or personal relationships that could have appeared to influence the work reported in this paper.

## Data Availability

All supporting data contributing to this study can be provided from the corresponding author on reasonable request.
