# Supplementary material for: Association of MRI indexes of glymphatic system with brain atrophy and cognitive impairment in cerebral small vessel disease
Source: Neuroimage Clin. 2026 Jan 18;49:103951. doi: 10.1016/j.nicl.2026.103951 (PMC12860371; doi:10.1016/j.nicl.2026.103951)
Supplement: Supplementary Data 1 [file mmc1.docx]

**Supplementary Information**

**Association of MRI Indexes of Glymphatic System with Brain Atrophy and Cognitive Impairment in Cerebral Small Vessel Disease**

Lulu Ai^1, †^, Zhiwei Li^1, †^, Chaojuan Huang^1^, Xia Zhou^1^, Xiaoqun Zhu^1, *^, Qiaoqiao Xu^2, *^, Zhongwu Sun^1, *^

^1^Department of Neurology, The First Affiliated Hospital of Anhui Medical University, Hefei, China.

^2^Department of Neurology, The Third Affiliated Hospital of Anhui Medical University (Hefei City First People’s Hospital), Hefei, China.

†These authors have contributed equally to this work.

*Corresponding author: Zhongwu Sun, Xiaoqun Zhu, and Qiaoqiao Xu.

*Corresponding author:

Zhongwu Sun and Xiaoqun Zhu, Department of Neurology, The First Affiliated Hospital of Anhui Medical University, 218 Jixi Road, Hefei, Anhui 230022, China. Tel.: +86 0551 62922328.

Qiaoqiao Xu, Department of Neurology, The Third Affiliated Hospital of Anhui Medical University (Hefei City First People’s Hospital), 390 Huaihe Road, Hefei, Anhui 230071, China. Tel.: +86 0551 62186777.

E-mails: sunzhwu@126.com (Zhongwu Sun), zxq_ayfy@163.com (Xiaoqun Zhu), [xqq7039@126.com](mailto:xqq7039@126.com) (Qiaoqiao Xu).

**Supplementary eMethod**

**MRI Data Acquisition**

We acquired MRI data using a 3.0-Tesla MR system (Discovery MR750w, General Electric, Milwaukee, WI, USA) with a 24-channel head coil. To reduce scanning noise, participants wore earplugs and foam padding restricted head movement. Simultaneously, participants were instructed to remain awake with eyes closed in a relaxed state. High resolution three-dimensional T1-weighted (3D-T1) images were acquired through a brain volume (BRAVO) sequence with the following parameters: slice thickness = 1.0 mm, repetition time (TR) = 8.464 ms, echo time (TE) = 3.248 ms, inversion time (TI) = 450 ms, flip angle (FA) = 12°, field of view (FOV) = 256 mm × 256 mm, matrix size = 256 × 256, slice thickness = 1 mm without gap, 188 sagittal slices and acquisition time = 296 s. T2-weighted images (T2WI) were acquired with the following parameters: TR = 5057 ms, TE = 127.7 ms, FA = 111°, FOV = 240 mm × 240 mm, matrix size = 512 × 512, and slice thickness = 5 mm. T2 fluid-attenuated inversion recovery (T2 FLAIR) images were acquired with the following parameters: TR = 9000 ms, TE = 119.84 ms, FA = 160◦, FOV = 225 mm × 225 mm, matrix size = 512 × 512, number of layers = 19, layer thickness = 7 mm and acquisition time = 1 min 57 s. Susceptibility-weighted imaging (SWI) images were acquired with the following parameters: TR = 45.4 ms, TE = 23.536 ms, FA = 20°, FOV = 240 mm × 240 mm, matrix size = 512 × 512, slice thickness = 1 mm, number of slices = 138, and acquisition time = 3 min 51 s. Diffusion tensor imaging (DTI) were acquired using a spin echo single-shot echo planar imaging (SE-SS-EPI) sequence with the following parameters: slice thickness = 3 mm without gap, TR = 10000 ms, TE = 74.2 ms, FOV = 256 mm × 256 mm, matrix size = 128 × 128, FA = 90°, 50 axial slices, 64 diffusion gradient directions (b = 1000 s/mm2) plus five b = 0 reference images, and acquisition time= 700 s.

**Abbreviation**

**Table *S1* Common English abbreviations**

| **Full name** | **Abbreviation** |
| --- | --- |
| Alzheimer’s disease | AD |
| Auditory verbal learning test | AVLT |
| Basal ganglia | BG |
| Body mass index | BMI |
| brain parenchymal fraction | BPF |
| Clinical dementia rating | CDR |
| Clock drawing task | CDT |
| Confidence interval | CI |
| Cerebral microbleeds | CMBs |
| Choroid plexus | CP |
| Cerebral small vessel disease | CSVD |
| Diffusion tensor imaging analysis along the perivascular space | DTI-ALPS |
| False discovery rate | FDR |
| White matter free water | FW-WM |
| Family-wise error | FWE |
| Gray matter volume | GMV |
| Healthy controls | HC |
| Mild cognitive impairment | MCI |
| Montreal cognitive assessment | MoCA |
| Magnetic resonance imaging | MRI |
| No cognitive impairment | NCI |
| Perivascular space volume fraction | PVS VF |
| Total intracranial volume | TIV |
| Trail making test-A | TMT-A |
| Trail making test-B | TMT-B |
| Vascular risk factors | VRFs |
| White matter hyperintensities | WMHs |

**Table *S2* Abbreviations of brain regions in figures**

| **Full name** | **Abbreviation** |
| --- | --- |
| left calcarine fissure | CAL.L |
| left middle temporal gyrus | MTG.L |
| left postcentral gyrus | PoCG.L |
| right calcarine fissure | CAL.R |
| right middle frontal gyrus | MFG.R |
| right precentral gyrus | PreCG.R |
| right superior temporal gyrus | STG.R |
| right thalamus | THA.R |

**Results**

**Table *S3* Presentation of raw glymphatic function metrics among different groups**

|  | HC  (N=40) | CSVD-NCI (N=52) | CSVD-MCI  (N=68) | *P* values |
| --- | --- | --- | --- | --- |
| CP volume | 0.871±0.25 | 1.030±0.29 | 1.16±0.32 | <0.001 |
| BG-PVS VF | 0.063±0.01 | 0.072±0.01 | 0.071±0.01 | <0.001 |
| Putamen-PVS VF | 0.022±0.01 | 0.026±0.01 | 0.027±0.01 | <0.001 |
| FW-WM fraction | 0.351±0.01 | 0.357±0.01 | 0.365±0.03 | 0.002 |
| DTI-ALPS index | 1.390±0.14 | 1.288±0.11 | 1.226±0.11 | <0.001 |

Abbreviation: HC, healthy control; CSVD, cerebral small vessel disease; NCI, no cognitive impairment; MCI, mild cognitive impairment; CP, choroid plexus; BG, basal ganglia; PVS VF, perivascular space volume fraction; FW-WM, white matter free water; DTI-ALPS, diffusion tensor imaging analysis along the perivascular space.

Table *S4* Coordinates of brain regions in the difference of GMV in HC, CSVD-NCI, and CSVD-MCI groups

| Group |  | Brain regions  (AAL) | Cluster size (voxel) | MNI coordinate | | | T values |
| --- | --- | --- | --- | --- | --- | --- | --- |
|  |  |  |  | X | Y | Z |  |
| HC>CSVD-MCI | Cluster 1 | CAL.L | 5856 | 28.5 | -55.5 | -34.5 | 4.80 |
|  | Cluster 2 | STG.R | 5581 | 52.5 | -15 | -30 | 5.00 |
|  | Cluster 3 | PreCG.R | 3664 | 63 | -16.5 | 28.5 | 5.30 |
|  | Cluster 4 | MTG.L | 3016 | -54 | -1.5 | -19.5 | 5.38 |
|  | Cluster 5 | MFG.R | 2158 | 36 | 40.5 | -13.5 | 6.73 |
|  | Cluster 6 | PoCG.L | 1311 | -60 | -3 | 15 | 4.81 |
|  | Cluster 7 | THA.R | 1162 | 18 | -73.5 | 10.5 | 4.19 |
|  | Cluster 8 | CAL.R | 961 | 12 | -73.5 | 10.5 | 4.49 |
| CSVD-NCI  >CSVD-MCI | Cluster 1 | STG.R | 1452 | 52.5 | 6 | -19.5 | 4.85 |

Abbreviation: GMV, gray matter volume; HC, healthy control; CSVD, cerebral small vessel disease; NCI, no cognitive impairment; MCI, mild cognitive impairment; AAL, anatomical automatic labelling; MNI, Montreal Neurological Institute; CAL.L, left calcarine fissure; STG.R, right superior temporal gyrus; PreCG.R, right precentral gyrus; MTG.L, left middle temporal gyrus; MFG.R, right middle frontal gyrus; PoCG.L, left postcentral gyrus; THA.R, right thalamus; CAL.R, right calcarine fissure.

Table *S5* Correlation analysis of glymphatic function metrics with demographic and vascular risk factors in CSVD group.

|  | CP volume | |  | | BG-PVS | |  | | Putamen-PVS | |  | FW-WM fraction | |  | DTI-ALPS index | |
| --- | --- | --- | --- | --- | --- | --- | --- | --- | --- | --- | --- | --- | --- | --- | --- | --- |
|  | β (SE) | *P* | |  | β (SE) | *P* | |  | β (SE) | *P* |  | β (SE) | *P* |  | β (SE) | *P* |
| Demographic characteristics | | | | | | | | | | | | | | | | |
| Age | 0.042 (0.013) | **0.002*** | |  | 0.029 (0.013) | **0.028*** | |  | 0.031 (0.013) | **0.022*** |  | 0.024 (0.014) | 0.088 |  | -0.032 (0.013) | **0.018*** |
| Sex | 0.333 (0.186) | 0.075 | |  | 0.533 (0.177) | **0.003*** | |  | 0.080 (0.184) | 0.665 |  | 0.485 (0.183) | **0.009*** |  | -0.599 (0.176) | **<0.001*** |
| Vascular risk factors | | | | | | | | | | | | | | | | |
| Hypertension | 0.058 (0.192) | 0.763 | |  | 0.010 (0.185) | 0.955 | |  | -0.129 (0.192) | 0.501 |  | -0.059 (0.193) | 0.761 |  | -0.219 (0.180) | 0.228 |
| Diabetes | 0.001 (0.239) | 0.995 | |  | 0.404 (0.233) | 0.086 | |  | 0.249 (0.244) | 0.310 |  | 0.327 (0.239) | 0.173 |  | -0.158 (0.231) | 0.496 |
| Hypercholesterolemia | -0.165 (0.204) | 0.420 | |  | 0.325 (0.196) | 0.099 | |  | 0.148 (0.206) | 0.473 |  | -0.345 (0.204) | 0.094 |  | 0.169 (0.194) | 0.384 |
| Smoking | -0.315 (0.238) | 0.188 | |  | 0.359 (0.231) | 0.122 | |  | 0.429 (0.239) | 0.075 |  | 0.160 (0.241) | 0.508 |  | -0.272 (0.227) | 0.233 |
| BMI | 0.073 (0.033) | **0.027*** | |  | 0.075 (0.031) | **0.019*** | |  | 0.072 (0.033) | **0.029*** |  | -0.011 (0.033) | 0.744 |  | -0.025 (0.032) | 0.435 |

Note: Age and sex were adjusted for other variables. Abbreviations: CSVD, cerebral small vessel disease; CP, choroid plexus; BG, basal ganglia; PVS VF, perivascular space volume fraction; FW-WM, white matter free water; DTI-ALPS, diffusion tensor imaging analysis along the perivascular space; BMI, body mass index; SE, standard error. * *P* < 0.05 after FDR correction.

Table *S6* Effect Sizes and Confidence Intervals for Sex Differences in Glymphatic Function Metrics

|  | Male  (Mean ± SD) | Female  (Mean ± SD) | Mean Difference  (95% CI) | Cohen's d  (95% CI) | P value |
| --- | --- | --- | --- | --- | --- |
| CP volume | 1.152 ± 0.336 | 1.048 ± 0.276 | 0.10 [-0.01, 0.22] | 0.35 [-0.02, 0.72] | 0.069 |
| BG-PVS VF | 0.074 ± 0.008 | 0.069 ± 0.008 | 0.004 [0.001, 0.01] | 0.49 [0.13, 0.86] | **0.009*** |
| Putamen-PVS VF | 0.027 ± 0.007 | 0.027 ± 0.007 | 0.0003 [-0.002, 0.002] | 0.05 [-0.31, 0.41] | 0.798 |
| FW-WM fraction | 0.366 ± 0.024 | 0.356 ± 0.017 | 0.01 [0.001, 0.017] | 0.46 [0.09, 0.83] | **0.017*** |
| DTI-ALPS index | 1.220 ± 0.108 | 1.287 ± 0.107 | -0.06 [-0.10, -0.02] | -0.59 [-0.96, -0.22] | **0.002*** |

Note: Data are presented as mean ± standard deviation. Mean difference, 95% CI and P-value are from ANCOVA models adjusted for age and VRFs. Cohen's d interpretation: |d| < 0.2 (negligible), 0.2-0.5 (small), 0.5-0.8 (medium), ≥0.8 (large). Bold indicates statistical significance at *P* < 0.05.* Abbreviations: CP, choroid plexus; BG, basal ganglia; PVS VF, perivascular space volume fraction; FW-WM, white matter free water; DTI-ALPS, diffusion tensor imaging analysis along the perivascular space; SD, standard deviation; CI, confidence interval.

Table *S7* ****Interactive effects of Sex and group on**** glymphatic function metrics ****in Patients with CSVD****

|  | Group | Male Mean (SD) | Female Mean (SD) | Sex  Main Effect  F(p) | Group  Main Effect F(p) | Sex x Group  Interaction F(p) |
| --- | --- | --- | --- | --- | --- | --- |
| CP volume | NCI | 1.03 (0.31) | 1.03 (0.27) | 4.03 (0.047)* | 3.77 (0.055) | 2.31 (0.131) |
|  | MCI | 1.26 (0.33) | 1.06 (0.28) |  |  |  |
| BG-PVS VF | NCI | 7.25 (0.65) | 7.10 (0.51) | 6.82 (0.010)** | 0.32 (0.571) | 2.69 (0.104) |
|  | MCI | 7.49 (0.98) | 6.80 (0.96) |  |  |  |
| Putamen-PVS VF | NCI | 2.58 (0.64) | 2.63 (0.57) | 0.09 (0.760) | 0.62 (0.432) | 0.30 (0.584) |
|  | MCI | 2.81 (0.67) | 2.67 (0.79) |  |  |  |
| FW-WM fraction | NCI | 36.0 (1.4) | 35.5 (1.2) | 6.67 (0.011)* | 3.26 (0.074) | 1.55 (0.215) |
|  | MCI | 37.3 (2.8) | 35.7 (2.0) |  |  |  |
| DTI-ALPS index | NCI | 1.25 (0.11) | 1.33 (0.11) | 11.89 (<0.001)*** | 8.94 (0.003)** | 0.13 (0.724) |
|  | MCI | 1.19 (0.10) | 1.26 (0.10) |  |  |  |

Note: Data are presented as mean (standard deviation). Two-way ANCOVA models were conducted with sex and group as independent variables, controlling for age and VRFs. F statistics and p-values are reported for main effects and interactions. Bold indicates statistical significance at *p* < 0.05.* Abbreviations: CP, choroid plexus; BG, basal ganglia; PVS VF, perivascular space volume fraction; FW-WM, white matter free water; DTI-ALPS, diffusion tensor imaging analysis along the perivascular space; SD, standard deviation.

Table *S8* Interactive effects of sex and age on glymphatic function metrics in patients with CSVD

|  | Male Mean (SD) | Female Mean (SD) | Age Effect (per year)  Slope (β) | Sex  Main Effect F(p) | Age  Main Effect F(p) | Sex x Age Interaction  F(p) |
| --- | --- | --- | --- | --- | --- | --- |
| CP volume | 1.15 (0.34) | 1.05 (0.28) | +0.018** | 3.41 (0.067) | 9.40 (0.002)** | 1.75 (0.188) |
| BG-PVS VF | 7.38 (0.85) | 6.93 (0.82) | +0.033* | 7.34 (0.008)** | 4.27 (0.041)* | 0.54 (0.463) |
| Putamen-PVS VF | 2.71 (0.66) | 2.65 (0.70) | 0.023 | 0.09 (0.763) | 2.96 (0.088) | 0.10 (0.748) |
| FW-WM fraction | 0.366 (0.024) | 0.356 (0.017) | 0.00067 | 6.14 (0.015)* | 2.81 (0.097) | 0.42 (0.518) |
| DTI-ALPS index | 1.22 (0.11) | 1.29 (0.11) | -0.0047* | 10.61 (0.001)** | 5.17 (0.024)* | 0.80 (0.373) |

Note: Data are presented as mean (standard deviation). Linear regression models were conducted with sex, age (centered at mean), and their interaction as independent variables, controlling for VRFs. Age slope (beta coefficient) represents the change in imaging marker per 1-year increase in age. F statistics and p-values are reported for main effects and interactions. Bold indicates statistical significance at *p* < 0.05.* Abbreviations: CP, choroid plexus; BG, basal ganglia; PVS VF, perivascular space volume fraction; FW-WM, white matter free water; DTI-ALPS, diffusion tensor imaging analysis along the perivascular space; SD, standard deviation.

Table *S9* Partial correlation analysis of glymphatic function metrics with brain GMV in CSVD group

|  | CP volume | | |  | BG-PVS | | |  | Putamen-PVS | | |  | FW-WM fraction | | |  | DTI-ALPS index | | |
| --- | --- | --- | --- | --- | --- | --- | --- | --- | --- | --- | --- | --- | --- | --- | --- | --- | --- | --- | --- |
|  | r | *P* | *P_FDR_* |  | r | *P* | *P_FDR_* |  | r | *P* | *P_FDR_* |  | r | *P* | *P_FDR_* |  | r | *P* | *P_FDR_* |
| CAL.L | -0.418 | 0.000 | 0.000 |  | -0.015 | 0.253 | 0.328 |  | -0.229 | 0.015 | 0.042 |  | -0.247 | 0.011 | 0.032 |  | 0.119 | 0.027 | 0.062 |
| CAL.R | -0.265 | 0.006 | 0.021 |  | -0.262 | 0.006 | 0.020 |  | -0.369 | 0.000 | 0.001 |  | -0.320 | 0.001 | 0.007 |  | 0.328 | 0.000 | 0.004 |
| MTG.L | -0.292 | 0.002 | 0.013 |  | -0.093 | 0.164 | 0.228 |  | -0.288 | 0.002 | 0.013 |  | -0.390 | 0.000 | 0.001 |  | 0.181 | 0.022 | 0.058 |
| MFG.R | -0.340 | 0.000 | 0.004 |  | 0.112 | 0.562 | 0.633 |  | -0.161 | 0.069 | 0.130 |  | -0.311 | 0.001 | 0.008 |  | 0.108 | 0.076 | 0.137 |
| PoCG.L | -0.471 | 0.000 | 0.000 |  | -0.096 | 0.163 | 0.228 |  | -0.361 | 0.000 | 0.002 |  | -0.310 | 0.001 | 0.008 |  | 0.282 | 0.003 | 0.013 |
| PreCG.R | -0.445 | 0.000 | 0.000 |  | 0.041 | 0.871 | 0.891 |  | -0.189 | 0.035 | 0.071 |  | -0.315 | 0.001 | 0.007 |  | 0.045 | 0.318 | 0.398 |
| STG.R | -0.358 | 0.000 | 0.002 |  | -0.031 | 0.148 | 0.217 |  | -0.280 | 0.003 | 0.014 |  | -0.250 | 0.010 | 0.030 |  | 0.351 | 0.000 | 0.002 |
| THA.R | -0.120 | 0.069 | 0.130 |  | -0.046 | 0.193 | 0.261 |  | -0.040 | 0.638 | 0.693 |  | -0.095 | 0.190 | 0.259 |  | 0.096 | 0.083 | 0.142 |
| BPF | -0.624 | 0.000 | 0.000 |  | -0.259 | 0.000 | 0.021 |  | -0.339 | 0.000 | 0.003 |  | -0.276 | 0.004 | 0.016 |  | 0.306 | 0.001 | 0.007 |

Note: Partial correlation analysis of glymphatic function metrics with brain GMV in CSVD group, controlling for demographics, VRFs, and TIV. Abbreviation: GMV, gray matter volume; CSVD, cerebral small vessel disease; CP, choroid plexus; BG, basal ganglia; PVS VF, perivascular space volume fraction; FW-WM, white matter free water; DTI-ALPS, diffusion tensor imaging analysis along the perivascular space; CAL.L, left calcarine fissure; CAL.R, right calcarine fissure; MTG.L, left middle temporal gyrus; MFG.R, right middle frontal gyrus; PoCG.L, left postcentral gyrus; PreCG.R, right precentral gyrus; STG.R, right superior temporal gyrus; THA.R, right thalamus; BPF, brain parenchymal fraction; VRFs, vascular risk factors; TIV: total intracranial volume; FDR, false discovery rate.

Table *S10* Partial correlation analysis of glymphatic function metrics with cognition in CSVD group

|  | CP volume | | |  | BG-PVS | | |  | Putamen-PVS | | |  | FW-WM fraction | | |  | DTI-ALPS index | | |
| --- | --- | --- | --- | --- | --- | --- | --- | --- | --- | --- | --- | --- | --- | --- | --- | --- | --- | --- | --- |
|  | r | *P* | *P_FDR_* |  | r | *P* | *P_FDR_* |  | r | *P* | *P_FDR_* |  | r | *P* | *P_FDR_* |  | r | *P* | *P_FDR_* |
| Global cognitive function | -0.197 | 0.161 | 0.228 |  | -0.189 | 0.227 | 0.300 |  | -0.284 | 0.031 | 0.066 |  | -0.221 | 0.121 | 0.193 |  | 0.328 | 0.000 | 0.004 |
| Memory function | -0.127 | 0.447 | 0.529 |  | 0.101 | 0.119 | 0.193 |  | -0.026 | 0.811 | 0.849 |  | -0.265 | 0.007 | 0.022 |  | -0.020 | 0.487 | 0.553 |
| Executive function | -0.247 | 0.025 | 0.061 |  | 0.068 | 0.264 | 0.336 |  | -0.033 | 0.914 | 0.914 |  | -0.137 | 0.453 | 0.531 |  | 0.258 | 0.030 | 0.066 |
| Processing speed | -0.191 | 0.078 | 0.138 |  | 0.061 | 0.324 | 0.401 |  | -0.058 | 0.845 | 0.877 |  | -0.074 | 0.870 | 0.891 |  | 0.232 | 0.015 | 0.042 |
| Language function | 0.000 | 0.701 | 0.751 |  | 0.170 | 0.035 | 0.071 |  | 0.057 | 0.395 | 0.472 |  | -0.298 | 0.002 | 0.013 |  | 0.053 | 0.716 | 0.761 |
| Visuospatial function | -0.214 | 0.082 | 0.142 |  | -0.132 | 0.331 | 0.406 |  | -0.165 | 0.256 | 0.329 |  | -0.284 | 0.004 | 0.016 |  | 0.286 | 0.003 | 0.013 |

Note: Partial correlation analysis of glymphatic function metrics with cognition in CSVD group, controlling for demographics, VRFs, and neuroimaging markers of CSVD. Abbreviation: CSVD, cerebral small vessel disease; CP, choroid plexus; BG, basal ganglia; PVS VF, perivascular space volume fraction; FW-WM, white matter free water; DTI-ALPS, diffusion tensor imaging analysis along the perivascular space; VRFs, vascular risk factors; FDR, false discovery rate.

Table *S11* Partial correlation analysis of brain GMV with cognition in CSVD group

|  | Global cognitive function | | |  | Memory function | | |  | Executive function | | |  | Processing speed | | |  | Language function | | |  | Visuospatial function | | |
| --- | --- | --- | --- | --- | --- | --- | --- | --- | --- | --- | --- | --- | --- | --- | --- | --- | --- | --- | --- | --- | --- | --- | --- |
|  | r | *P* | *P_FDR_* |  | r | *P* | *P_FDR_* |  | r | *P* | *P_FDR_* |  | r | *P* | *P_FDR_* |  | r | *P* | *P_FDR_* |  | r | *P* | *P_FDR_* |
| CAL.L | 0.269 | 0.132 | 0.200 |  | 0.247 | 0.026 | 0.061 |  | 0.184 | 0.248 | 0.325 |  | 0.142 | 0.203 | 0.272 |  | 0.034 | 0.880 | 0.893 |  | 0.112 | 0.347 | 0.422 |
| CAL.R | 0.349 | 0.093 | 0.155 |  | 0.239 | 0.085 | 0.144 |  | 0.175 | 0.486 | 0.553 |  | 0.139 | 0.693 | 0.202 |  | 0.088 | 0.624 | 0.685 |  | 0.275 | 0.065 | 0.127 |
| MTG.L | 0.360 | 0.128 | 0.196 |  | 0.276 | 0.023 | 0.058 |  | 0.303 | 0.031 | 0.066 |  | 0.248 | 0.126 | 0.196 |  | 0.185 | 0.106 | 0.174 |  | 0.343 | 0.023 | 0.058 |
| MFG.R | 0.276 | 0.004 | 0.016 |  | 0.251 | 0.009 | 0.028 |  | 0.210 | 0.079 | 0.138 |  | 0.080 | 0.485 | 0.553 |  | 0.225 | 0.025 | 0.061 |  | 0.133 | 0.142 | 0.221 |
| PoCG.L | 0.269 | 0.005 | 0.018 |  | 0.270 | 0.005 | 0.018 |  | 0.240 | 0.012 | 0.035 |  | 0.284 | 0.003 | 0.014 |  | 0.030 | 0.783 | 0.825 |  | 0.273 | 0.004 | 0.017 |
| PreCG.R | 0.335 | 0.071 | 0.131 |  | 0.211 | 0.133 | 0.200 |  | 0.254 | 0.068 | 0.130 |  | 0.287 | 0.003 | 0.013 |  | 0.166 | 0.150 | 0.218 |  | 0.220 | 0.122 | 0.193 |
| STG.R | 0.353 | 0.000 | 0.002 |  | 0.252 | 0.009 | 0.028 |  | 0.272 | 0.034 | 0.071 |  | 0.205 | 0.054 | 0.108 |  | 0.276 | 0.004 | 0.016 |  | 0.313 | 0.001 | 0.007 |
| THA.R | 0.311 | 0.075 | 0.137 |  | 0.263 | 0.029 | 0.064 |  | 0.161 | 0.472 | 0.549 |  | 0.148 | 0.313 | 0.395 |  | 0.045 | 0.913 | 0.914 |  | 0.245 | 0.011 | 0.032 |
| BPF | 0.192 | 0.376 | 0.453 |  | 0.164 | 0.178 | 0.245 |  | 0.193 | 0.158 | 0.227 |  | 0.274 | 0.023 | 0.058 |  | -0.008 | 0.612 | 0.678 |  | 0.279 | 0.053 | 0.107 |

Note: Partial correlation analysis of brain GMV with cognition in CSVD group, controlling for demographics, VRFs, neuroimaging markers of CSVD, and TIV. Abbreviation: GMV, gray matter volume; CSVD, cerebral small vessel disease; CAL.L, left calcarine fissure; CAL.R, right calcarine fissure; MTG.L, left middle temporal gyrus; MFG.R, right middle frontal gyrus; PoCG.L, left postcentral gyrus; PreCG.R, right precentral gyrus; STG.R, right superior temporal gyrus; THA.R, right thalamus; BPF, brain parenchymal fraction; VRFs, vascular risk factors; TIV: total intracranial volume; FDR, false discovery rate.

**
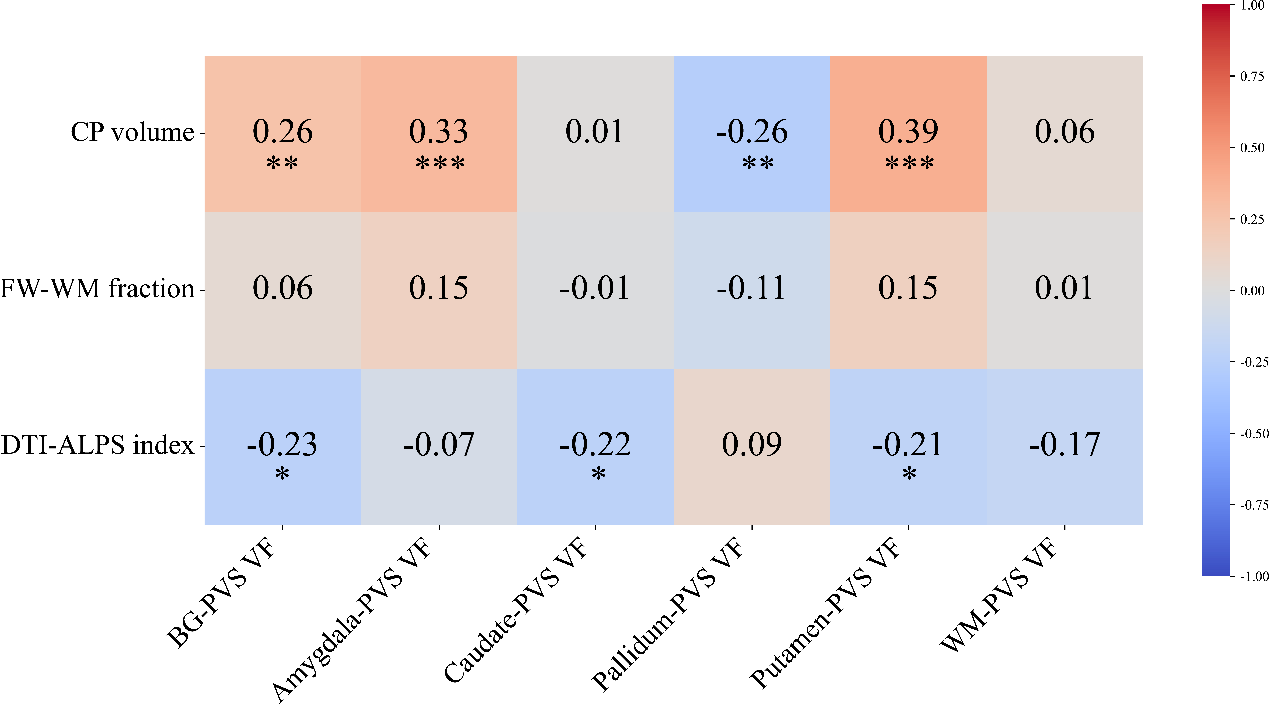
**

**Figure *S1*** Correlation analysis of glymphatic function metrics with whiter matter PVS and BG-PVS (including subregions: Amygdala, Caudate, Pallidum, and Putamen) in CSVD group. Abbreviation: CSVD, cerebral small vessel disease; CP, choroid plexus; FW-WM, white matter free water; DTI-ALPS, diffusion tensor imaging analysis along the perivascular space; BG, basal ganglia; PVS VF, perivascular space volume fraction; WM, white matter. **P* < 0.05, ***P* < 0.01, ****P* < 0.001.


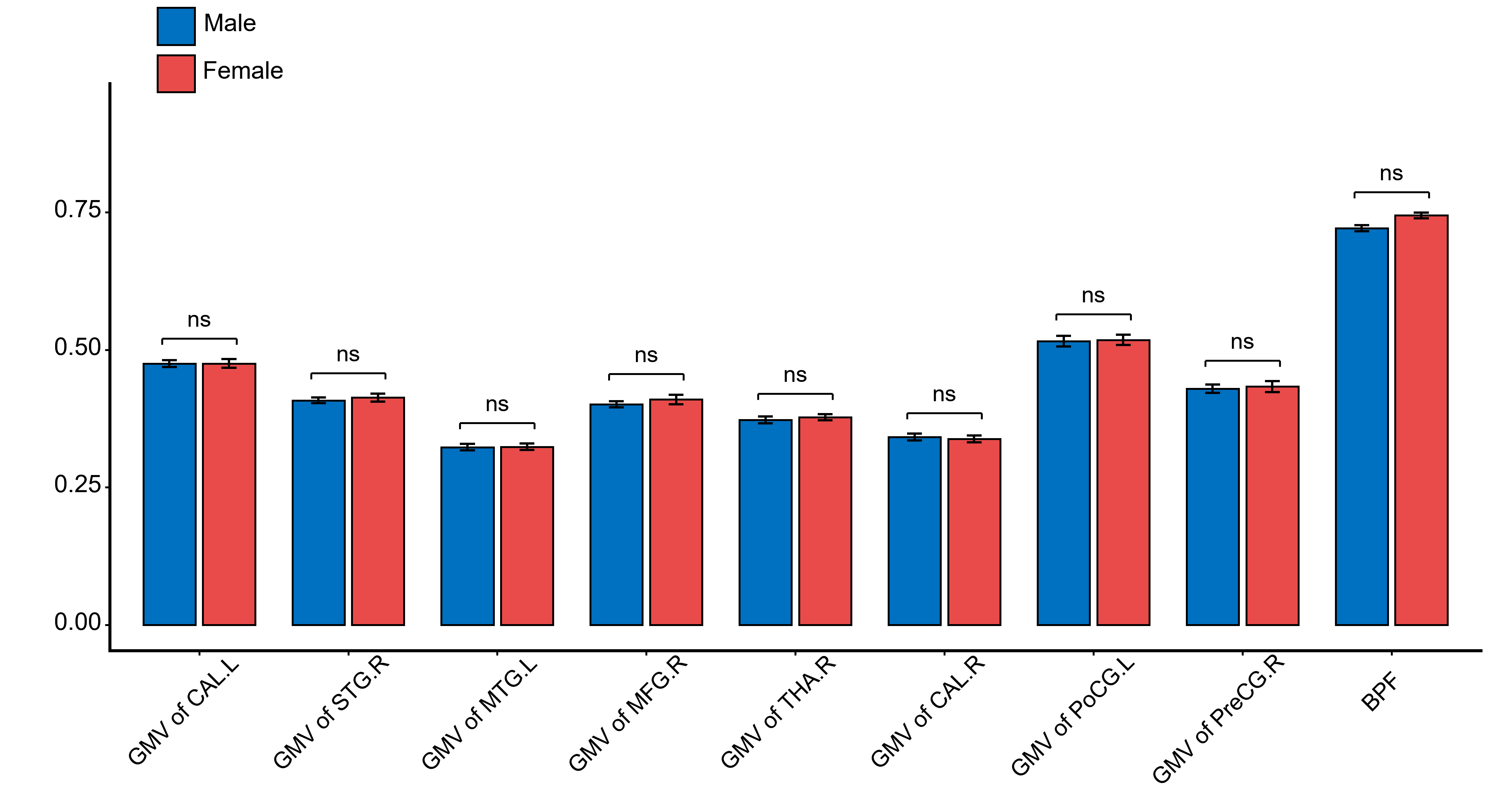


**Figure *S2*** Comparison of sex distribution difference in brain atrophy metrics among CSVD patients, with controlled for age, VRFs (hypertension, diabetes, hypercholesterolemia, smoking, and BMI), and TIV. Abbreviations: CSVD, cerebral small vessel disease; VRFs, vascular risk factors; BMI, body mass index; TIV, total intracranial volume; GMV, gray matter volume; CAL.L, left calcarine fissure; CAL.R, right calcarine fissure; MTG.L, left middle temporal gyrus; MFG.R, right middle frontal gyrus; PoCG.L, left postcentral gyrus; PreCG.R, right precentral gyrus; STG.R, right superior temporal gyrus; THA.R, right thalamus; BPF, brain parenchymal fraction. ns not statistically significant, **P* < 0.05, ***P* < 0.01, ****P* < 0.001.


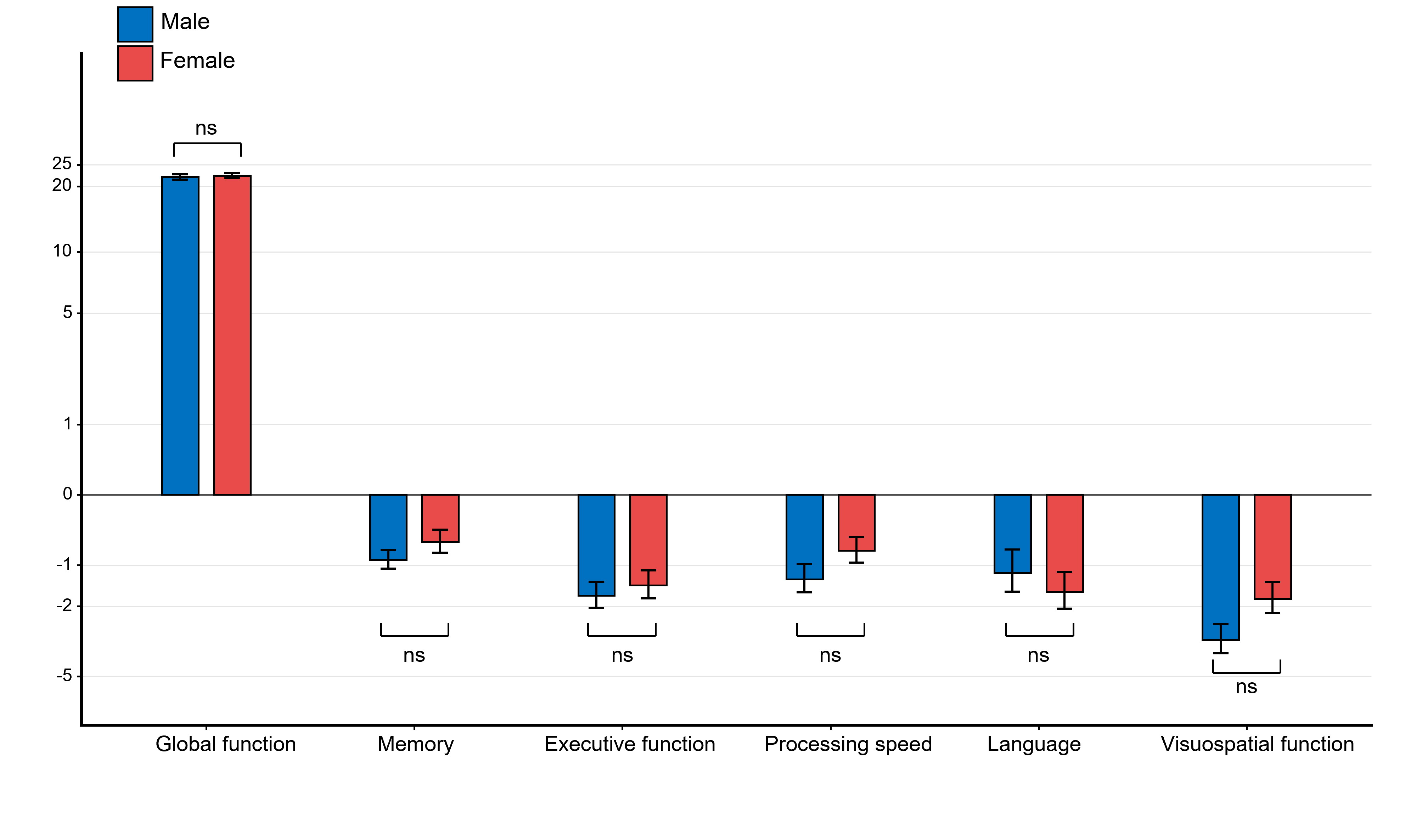


**Figure *S3*** Comparison of sex distribution difference in cognitive function among CSVD patients, with controlled for age, VRFs (hypertension, diabetes, hypercholesterolemia, smoking, and BMI), and CSVD neuroimaging markers (WMH Fazekas scores, lacunes, CMBs). Abbreviations: CSVD, cerebral small vessel disease; VRFs, vascular risk factors; BMI, body mass index; WMH, white matter hyperintensity; CMBs, cerebral microbleeds. ns not statistically significant, **P* < 0.05, ***P* < 0.01, ****P* < 0.001.
